# One step at a time: Physical activity is linked to positive interpretations of ambiguity

**DOI:** 10.1371/journal.pone.0225106

**Published:** 2019-11-14

**Authors:** Maital Neta, Nicholas R. Harp, Daniel J. Henley, Safiya E. Beckford, Karsten Koehler

**Affiliations:** 1 Department of Psychology, University of Nebraska-Lincoln, Lincoln, NE, United States of America; 2 Center for Brain, Biology and Behavior, University of Nebraska-Lincoln, Lincoln, NE, United States of America; 3 Department of Advertising and Public Relations, Michigan State University, East Lansing, Michigan, United States of America; 4 Department of Nutrition and Health Sciences, University of Nebraska-Lincoln, Lincoln, NE, United States of America; 5 Department of Sport and Health Sciences, Technical University of Munich, Munich, Germany; Universite Cote d'Azur, FRANCE

## Abstract

**Background:**

Extensive research has established a clear positive relationship between physical activity (PA), even in small amounts, and psychological well-being, including benefits for emotional and mental health (e.g., decreased depression). However, little research has examined the relationship between PA and decision-making within emotionally ambiguous contexts. The purpose of the present cross-sectional study was to examine the relationship between reported amount and intensity of PA and interpretations of emotional ambiguity.

**Methods:**

Adults (n = 611) recruited through Amazon’s Mechanical Turk were assessed on their interpretations of ambiguous and clear (unambiguous) emotional stimuli and reported habitual PA and exercise.

**Results:**

More positive ratings of ambiguity were associated with greater amount of vigorous activity (p = .002), but not with moderate activity (p = .826) or walking (p = .673). Subsequent analyses demonstrated that this relationship between vigorous PA and positive interpretations of ambiguity was most pronounced when comparing individuals who reported any amount of vigorous PA to those who reported no vigorous activity at all.

**Conclusions:**

Our findings suggest that higher amounts of vigorous, but not moderate, PA are associated with more positive interpretations of ambiguity, and that even small amounts of PA seem to be sufficient to promote this more positive valence bias when compared to individuals conducting no vigorous PA at all. Future work should examine the longitudinal effects of PA among individuals participating in structured activity programs.

## Introduction

The beneficial effects of a physically active lifestyle are well documented [1]. Indeed, extensive research has examined the favorable impact of habitual physical activity (PA) and exercise (i.e., a specific subset of PA with the goal of improving or maintaining health- or skill-related physical fitness [2]) on both cognitive control [3–4] and emotional well-being [5–8]. Much of this work has found that there are cognitive and emotional benefits of both PA and exercise in adults [9–11] and in children (e.g., [10, 12]). While it is challenging to disentangle the effects of these closely related constructs, the effects of PA, more broadly, and exercise, more specifically, are discussed below. It is worth noting that much of the research examining the effects of PA and exercise focuses on two primary components: the amount, which can further be divided into frequency and duration, and the intensity of PA or exercise [13]. Research related to PA more broadly has found that greater frequency of PA is associated with better inhibition (i.e., faster responses in a flanker task of attention; [10]), and more intense PA has been linked to better overall cognitive function, including better performance on measures of processing speed, memory, and mental flexibility [14].

In research focusing on the effects of exercise, voluntary exercise was found to induce widespread neurobiological adaptations associated with improved learning and memory [15] and may even serve as an effective intervention for attention-deficit/hyperactivity disorder [16]. Other work found that an acute exercise bout improves executive processing [5], which may contribute to increased cognitive control. Further, there are findings demonstrating that the amount of exercise is positively associated with cognitive benefits [6–7]. For example, there is accumulating evidence that more frequent exercise results in a range of cognitive benefits, including improvements in working memory [6], selective attention, and inhibitory control [6–7]. Also, exercise duration within an acute exercise bout significantly moderates the effects for cognitive outcomes, with longer durations (i.e., greater than 20 minutes) resulting in stronger effects [7]. With regards to intensity, a recent meta-analysis revealed that, in acute exercise interventions, intensity moderates the relationship between exercise and cognitive performance, with larger effect sizes after high, compared to moderate or low, intensity exercise [7]. Higher intensity exercise has also been found to support larger improvements in inhibitory control [17], and is more effective than moderate intensity exercise in improving executive functioning in that it results in a more sustained improvement post-exercise [18].

While there is extensive research linking PA and exercise to cognitive improvement, there are also notable effects on emotion processing [5, 9, 19–21]. For example, higher volumes, which includes both amount and intensity, of PA and exercise predict lower symptoms of depression [9, 19–20, 22] and anxiety [22], and reduce negative affect (i.e., frustration) related to difficult tasks (i.e., task-switching or memory tasks; [5]). With regards to effects of PA more broadly, some research has demonstrated that chronic physical activity mitigates the harmful consequences of an acute stressor [15]. Also, greater amount (in terms of duration) and intensity of PA are associated with more positive affect in participants with major depressive disorder [20]. And research examining the more specific effects of exercise found that exercise acutely induces more positive affect [21] regardless of intensity [23], and leads to physiological changes capable of producing antidepressant effects [11]. Further, regular exercise has been shown to improve mood and decrease state anxiety [24]. Other work found that, after exercise, participants rated unpleasant images as less emotionally arousing, but there was no effect on ratings of emotional valence (i.e., positive versus negative [8]). One potential explanation for this pattern of findings is that clearly valenced stimuli (e.g., images with a clear negative meaning) are not malleable to these PA- or exercise-related effects, given that it is difficult to change the interpretation of a negative image–e.g., a picture of a child crying–to be positive. To date, there has been no research testing the effects of PA and/or exercise on valence interpretations of emotionally ambiguous stimuli that are more malleable [25–26].

A separate line of research has demonstrated that interpretations of ambiguity can reveal important individual differences in emotion processing and coping strategies, and deficiencies in these skills can have widespread effects on mental and social functioning. This work has examined interpretations of facial expressions, as these social cues can inform and predict important outcomes in social environments [27]. In other words, appropriate interpretations of facial expressions are imperative for facilitating successful social interactions. When these facial cues are presented in the absence of contextual information that could disambiguate their meaning, perceivers must rely on previous experiences or biases to guide their interpretations. For example, although some facial expressions convey a clear meaning (e.g., angry faces convey a negative meaning and happy faces convey a positive one), other expressions (i.e., surprised) are more ambiguous in that they convey both positive (e.g., unexpected visit from an old friend) and negative (e.g., witnessing a car accident) meanings. A growing body of work has found that there is a wide range of variability in valence interpretations of emotionally ambiguous images (e.g., surprised faces), and this variability is represented as one’s valence bias, or the tendency to interpret emotional ambiguity as positive or negative [25, 28–32]. The valence bias represents a stable, trait-like difference between people, as it is stable across time [29] and generalizes to non-face emotionally ambiguous stimuli [28].

Our working model suggests that, despite these individual differences in valence bias, the initial perception of ambiguity is negative in all people [25–26, 30–32]. This *initial negativity hypothesis* posits that a positive valence bias arises in individuals that regulate or override this initial negativity in order to produce a positive interpretation, putatively via a mechanism similar to cognitive reappraisal (an emotion regulation strategy requiring mental flexibility and involving the reinterpretation of emotional stimuli to reduce the emotional impact; e.g., reinterpreting negative images to have a less negative–or even a positive–meaning [33–34]). Interestingly, this mechanism of emotion regulation is also associated with greater psychological well-being [35–37].

Consistent with our *initial negativity hypothesis*, our ongoing work shows preliminary evidence that a positive valence bias may be associated with increased well-being and resilience in the face of potential threats (see also [38–39]). In turn, other research has found similar effects of PA and exercise; as little as one hour of exercise enhances mood [40], decreases anxiety [41], and improves emotion regulation ability, which boosts psychological well-being [42]. While there is clear evidence that habitual exercise is associated with enhanced emotion regulation success (cognitive reappraisal, specifically [43]), there is accumulating evidence of a reliable relationship between PA and psychological well-being that transcends the quantity and intensity of activity [44]. For instance, a recent review reported that people who were physically active for only 10 minutes per day, or only once or twice per week, reported greater happiness than those who were sedentary [44].

Although previous work has examined the effects of PA/exercise and valence bias, separately, on emotion regulation (including cognitive reappraisal; [33–34, 42]), there have been no studies examining the direct relationship between PA and valence bias. This study aims to address this important gap in the literature. Given that PA and exercise are associated with better emotion regulation [43], and that enhanced emotion regulation (reappraisal) ability is associated with a more positive valence bias [33], we would predict that PA/exercise are linked with a positive valence bias.

### The present study

Here, we set out to extend previous work characterizing the relationship between PA and psychological well-being and affect by demonstrating a link between greater PA/exercise and more positive interpretations of ambiguity. Specifically, this work includes three goals. First, we seek to establish a link between total PA volume, which conflates amount and intensity, and a positive valence bias. Based on the previous literature associating PA and enhanced emotion regulation and mental flexibility, we predict a positive association between the PA *volume* and a positive bias. Because it is possible that the relationship with PA is modulated by *intensity*, our second goal is to examine the relationship for the amount of PA separately for vigorous and moderate intensities. In light of previous literature focusing on vigorous PA and demonstrating a benefit on psychological well-being [45–46] and the fact most (albeit not all) exercise is conducted at intensities corresponding to vigorous PA [47], we predict that individuals who conduct more vigorous (but not moderate) PA will show a more positive valence bias, and we will test this also as a function of duration and frequency of vigorous activity performed. Lastly, we will explore the relationship between valence bias and exercise per se. Although frequently used interchangeably in the literature, PA and exercise describe different concepts: PA refers to any bodily movement produced by skeletal muscles that results in energy expenditure and includes occupational and household activities, transportation by bike or foot, and sports and conditioning [2]. In contrast, exercise represents a specific subset of PA that is planned, structured and repetitive and has a goal of improving or maintaining health- or skill-related physical fitness, and includes virtually all conditioning and sports activities. Again, we predict that a greater exercise amount will be associated with a positive bias.

## Materials and methods

This study examined the cross-sectional relationship between valence bias, PA and exercise using Amazon’s Mechanical Turk (M-Turk). Utilizing M-Turk in research has been shown to be beneficial, particularly for individual difference studies such as this one, because the participants are significantly more demographically diverse than typical American college samples. Also, M-Turk data was found to meet acceptable psychometric standards such as high test-retest reliability [48].

### Participants

Participants aged 18 years and older were recruited through M-Turk (N = 611) for an online experiment. Thirty-five participants were excluded because they failed to provide accurate ratings of clearly valenced faces (angry, happy) on at least 60% of trials, consistent with exclusion criteria in previous work [25, 28–29]. Thirteen additional participants were excluded because their level of reported PA was outside of the feasible range (greater than 24 hours per day or seven days per week). The final sample included 563 participants (223 females). As expected from this M-Turk sample, these participants were demographically diverse with regards to age (18–69 years, average 32.6), education background (from having only some high school completed to having completed a doctorate), household income (<$10K/year to >$150K/year), and nationality (including countries in five continents; Table 1). The University of Nebraska-Lincoln’s Institutional Review Board approved all research protocols (approval #20150114791EP), and participants gave written informed consent prior to testing in accordance with the Declaration of Helsinki.

**Table 1:** Participant means and descriptive statistics M ± SD.

	All Participants (N = 563)
Age (years)	32.62 ± 8.15
Education	Some high school (1), high school diploma or GED (66), trade, technical, or vocational training (3), some college (116), associate’s (60), bachelor’s (298), master’s (0), PhD, medical, or law degree (5), professional degree (11), no response (3)
Race	African American (42), Asian (95), Hispanic (24), Other (6), Pacific Islander (1), White/Caucasian (395)
Income^a^	4.28 + 3.16
Walking (minutes/week)	214 + 390
Moderate Activity (minutes/week)	120 ± 233
Vigorous Activity (minutes/week)	122 ± 159
State Anxiety (STAI)	39.13 ± 11.96
Trait Anxiety (STAI)	40.34 ± 13.09
Ambiguous Ratings (% Negative)	56.8 ± 17.7

^a^Income ranged from 0 (less than $10,000) to 11 (more than $150,000).

### Stimuli

**Fig 1:**
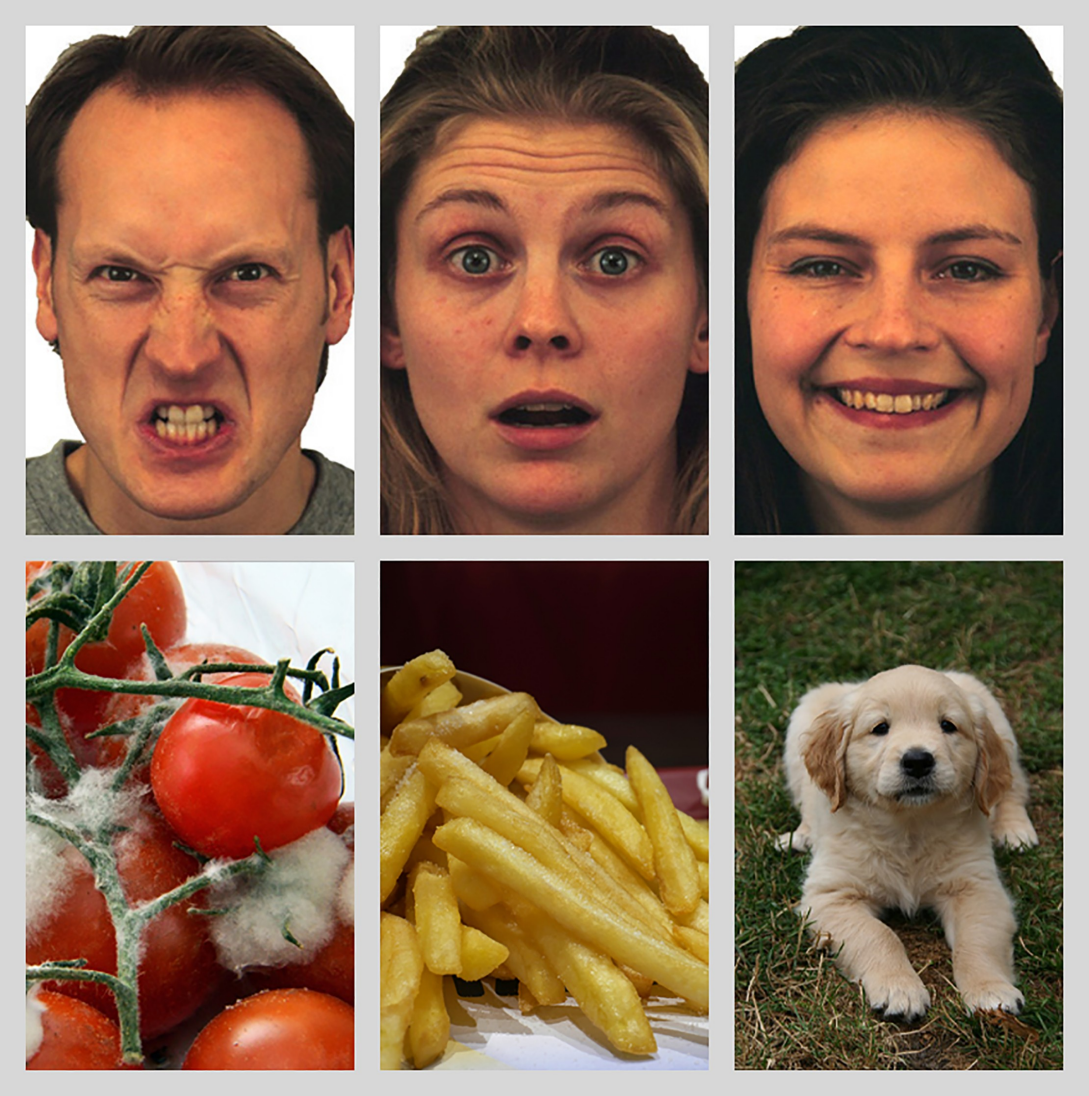
Negative, ambiguous, and positive images. Examples of clearly valenced and ambiguous images are shown. The selected faces come from the KDEF stimulus set and feature AF06HAS, AM10ANS, and AF30SUS. The additional scenes represent the types of IAPS stimuli used in our current study.

The stimuli were images of faces (48 pictures) and scenes (48 pictures) taken from previous work [18]. Each stimulus category (faces and scenes) included images with a clear (24 pictures: 12 negative, 12 positive) and ambiguous (24 pictures) valence (Fig 1). For the face stimuli, 34 unique face identities (17 women, 17 men) were selected from the NimStim standardized facial expressions stimulus set [49] and the averaged Karolinska Directed Emotional Faces database [50]. A subset of happy, angry, and surprised expressions was selected from each face identity to build the final stimulus set (i.e., not all expressions for each identity were used). The expressions were validated in prior work in which a separate set of participants labeled each expression [49]; only faces over 60% correctly labeled were included. The scenes were taken from the International Affective Picture System (IAPS [51]), and included images with a positive, negative and ambiguous valence, as defined in prior work [28]. Importantly, there was no significant difference in arousal ratings across the three conditions of scenes [28]. Given that the valence bias for faces and scenes were correlated (r = .293, p < .001), as in previous work [28], and that a combined measure of valence bias for both stimulus types would represent the bias in a more stable manner (i.e., findings will be more robust to the combined measure that generalizes across stimulus type than to either stimulus type alone), we used an average measure of ratings of ambiguous faces and scenes for a more robust measure of valence bias for each person.

### Paradigm

The experiment consisted of 4 alternating blocks of faces and IAPS (2 faces, 2 IAPS). Each block had 24 trials (12 clear valence, 12 ambiguous). The order of the pictures within a block was randomized across all participants. For each trial, participants first saw a fixation cross (500ms), then they saw a picture (500ms), and participants used their mouse to rate the picture as either positive or negative (two-alternative forced choice) by clicking on the appropriate response option, consistent with previous work [25, 28–29]. Participants were instructed to rate each image as fast as possible and go with their gut feelings. After completing this task, participants completed surveys assessing PA and exercise.

### Assessment of PA and exercise

The short form of the International Physical Activity Questionnaire (IPAQ [52]) was used to assess the participants PA volume (i.e., amount and intensity) over the course of the past seven days. The IPAQ assesses all components of PA, including occupational activities, transportation, household activities and recreation, sport, and leisure and includes both moderate and vigorous activities. Prior to completing the IPAQ, participants were given a general definition of exercise (“planned, structured, and repetitive physical activity done to promote health and fitness”), with examples (“walking, jogging, running, stair climbing, aerobics, water aerobics, cycling, rowing, swimming, weight training, etc.”), particularly delineated from sport activities (“requires effort and skill and is played according to rules, sport examples include: tennis, basketball, softball, football, golf, racquetball, soccer, etc.”) and leisure (“any activity done for enjoyment”). Consistent with previous work [53], these definitions and examples were provided to help participants tally all of their activities as part of PA, as participants are simply asked to provide one aggregate number for all PA rather than separately for exercise, sport and leisure. Participants then completed the IPAQ, with its specific instructions to “think about the activities you do at work, as part of your house and yard work, to get from place to place, and in your spare time for recreation, exercise or sport.” After completion of the IPAQ, participants were asked to specify their amount (i.e., hours per week) of exercise or sport activity in a typical week in order to separately explore for the relationship of valence bias with exercise.

In order to quantify the total PA volume across all activities and intensities, IPAQ data was converted to metabolic equivalent minutes (METmins), a simple physiological measure of the energy expended during PA [54]. METmins were calculated as the activity duration (in minutes per day) multiplied by the frequency (in days per week) and a MET factor of 4 for moderate intensity and walking and a factor of 8 for vigorous intensity, in accordance with the IPAQ analysis manual [52]. This procedure is in accordance with current physical activity guidelines, as well as other research [55], which recommend an interchangeable amount of moderate or vigorous physical activity, so long as moderate activity duration is twice that of vigorous activity [46]. While METmins were used for analyses of total PA, subsequent analyses of unique PA intensities (i.e., vigorous, moderate, and walking) were completed with overall time (min/week) as a measure of amount as well as measures of frequency (days/week) and duration (min/day) to align with PA guidelines expressed in these units.

In order to minimize response bias (i.e., habitually more active individuals being more likely to participate in this study), the study advertisement did not include any information indicating that PA or exercise behaviors would be assessed. The language in the informed consent document was also held deliberately vague in order to not emphasize the assessment of PA and exercise, and the PA and exercise surveys were administered only at the end of the survey in efforts to minimize the impact of the PA assessment on previous tasks.

### Statistical analyses

First, ratings across the face and IAPS stimulus categories were averaged to create three conditions: negative (angry faces and negative IAPS), positive (happy faces and positive IAPS), and ambiguous (surprised faces and ambiguous IAPS). Next, percent negative ratings were calculated as the percent of trials an image was rated as negative out of the total number of trials for that condition (excluding omissions; see [29]). All analyses accounted for age and sex, and used a Bonferroni correction for multiple comparisons. Due to the small number of subjects that did not identify as either male or female, we used a binary sex variable and excluded those who did not indicate male or female sex (n = 7) from any analyses including sex.

The relationship between total PA volume (i.e., combining vigorous, moderate, and walking levels) and valence interpretations of images was examined by testing correlations between total PA (in METmins) and valence ratings of negative, positive, and ambiguously valenced images. All correlational analyses used Spearman correlations because Shapiro-Wilk tests of normality indicated that values for each variable were not normally distributed.

Next, we examined the relationship between valence interpretations of images and different intensities of PA, taking a more nuanced approach to relating the two variables. In other words, amounts of vigorous and moderate PA as well as walking, expressed in minutes per week, were correlated with ratings of the negative, positive, and ambiguous images. In these analyses, we also controlled for PA at the other intensity levels (e.g., controlling for moderate and walking in the analysis of vigorous PA). Following any significant effects with amount of PA at these different intensity levels, we ran post-hoc tests correlating PA in frequency (days per week) and duration (minutes per day) with valence ratings. Again, following any significant effects in these analyses, we examined the dose-dependent relationship between valence ratings and PA–for example, comparing individuals at adjacent levels of PA frequency (8 levels: 0–7 days per week) and duration (5 levels: 0 minutes, less than 30 minutes, 30–35 minutes, 36–59 minutes, and 60 or more minutes per day). For these analyses, data were modeled using linear regression and a series of dummy codes to test the significance of adding one day of vigorous activity (0 vs. 1, 1 vs. 2, etc.) or adding increasing minutes of activity (0 vs. < 30 minutes, etc.). Finally, we correlated exercise amount (in hours per week) with valence ratings of the three image conditions (negative, positive, and ambiguous). We conducted all analyses using R Statistical Software version 3.6.0 [56].

## Results

### Valence ratings

Participants rated negative images as negative (mean = 96.5% negative, SD = 4.9; range = 70.8–100), and positive images as positive (mean = 1.6% negative, SD = 3.5; range = 0–20.8). In contrast, there was a wide range of inter-subject variability in ratings of ambiguous images (mean = 56.8% negative, SD = 17.7; range = 2.1–95.8; S1 Fig), which represented the valence bias for each individual (Table 1).

### Association between valence ratings and total PA

Total PA, when expressed in METmins per week, was not significantly related to ratings of ambiguous images (r = -.074, p = .086; Bonferroni corrected significance is p < .0167), positive images (r = -.078, p = .072) or negative images (r = -.016, p = .710). Despite any effect here with total PA, our next goal was to explore the relationship between ratings and PA of varying intensities in minutes per week. Specifically, we predicted that these effects might be modulated by intensity, such that the relationship is particularly robust for vigorous PA.

### Association between valence ratings and PA intensity

We tested for independent relationships between vigorous, moderate, and walking PA (in minutes per week) and valence ratings of negative, positive, and ambiguous images. There was a significant negative correlation between vigorous PA and ratings of ambiguous images (r = -.145, p = .002; Bonferroni corrected significance is p < .0167), such that more vigorous PA was associated with more positive ratings of ambiguity. In contrast, there was no relationship between vigorous PA and ratings of positive (r = .009, p = .846) or negative images (r = -.085, p = .070). There was also no relationship between moderate PA or walking minutes per week and any of the image ratings (all p’s > .200 except for the relationship between walking minutes per week and more positive ratings of positive images, which did not survive correction for multiple comparisons, p = .075; Bonferroni corrected significance p < .0167).

To further probe this relationship between vigorous PA and ratings of ambiguity, we examined the relationship between vigorous PA in frequency (days per week) and duration (minutes per day) and ratings. The frequency of vigorous PA was negatively correlated with ambiguous ratings (r = -.137, p = .002; surviving Bonferroni correction p < .0167), such that more days of vigorous activity per week were associated with more positive ratings of ambiguously valenced images. A similar effect was found for duration of vigorous PA (r = -.144, p = .002; Bonferroni corrected significance p < .0167). A summary of these results is available in Table 2.

**Table 2:** Correlations summary.

Physical Activity Type	Image Type	Spearman’s rho	p-value
Total PA (MET minutes per week)	Ambiguous	-.074	.086
	Positive	-.078	.072
	Negative	-.016	.710
Vigorous PA (minutes per week)	Ambiguous	-.145	.002*
	Positive	.009	.846
	Negative	-.085	.070
Moderate PA (minutes per week)	Ambiguous	-.010	.827
	Positive	-.060	.201
	Negative	.041	.385
Walking (minutes per week)	Ambiguous	-.020	.674
	Positive	-.084	.075
	Negative	-.043	.366
Vigorous PA (days per week)	Ambiguous	-.137	.002*
Vigorous PA (minutes per day)	Ambiguous	-.144	.002*
Exercise (hours per week)	Ambiguous	-.157	< .001*
	Positive	-.064	.131
	Negative	.016	.711

*significant after Bonferroni correction for multiple comparisons

### Dose-dependent relationship between valence bias and vigorous PA

**Fig 2:**
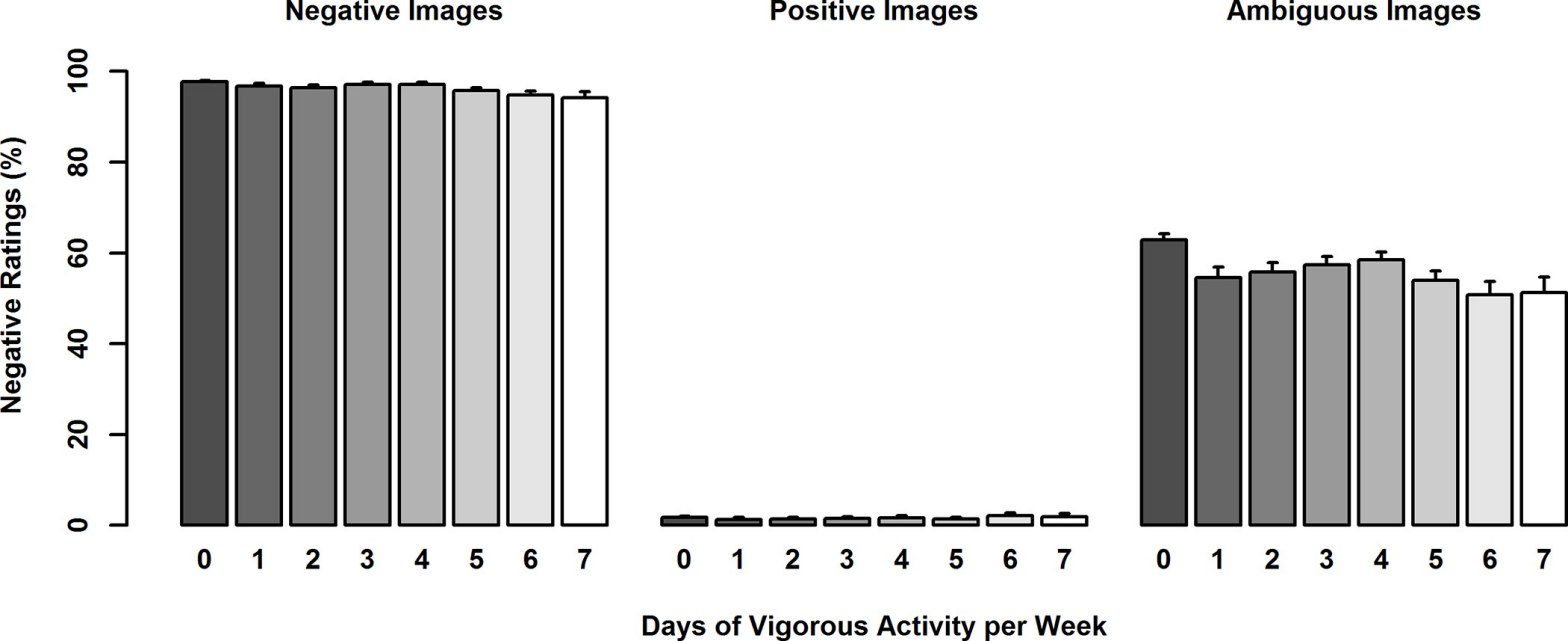
Percent negative ratings of emotional images as a function of vigorous activity in days per week. Ambiguous images were rated more positively by individuals that reported at least one day of vigorous activity per week as compared to those who reported no vigorous activity per week (p = .002; surviving a Bonferroni corrected significance p < .007). All other adjacency comparisons were not significant (p’s > .13).

A series of linear regressions showed significant effects of frequency of vigorous activity on ratings of ambiguity (8 frequency levels of vigorous PA: 0–7 days per week; F(9, 542) = 4.34, p < .001). When comparing adjacent frequency levels (i.e., comparing individuals who were physically inactive—0 days of vigorous activity—to those who were physically active just 1 day per week, then comparing 1 to 2 days, etc.), the only significant effects were observed between 0 and 1 days and only for ratings of ambiguity (p = .002; surviving a Bonferroni corrected significance p < .007), such that those engaging in vigorous PA for 1 day per week were more positive than those who were inactive; all other adjacency comparisons were not significant (p’s > .13; Fig 2). Sex contributed to the model, such that females rated ambiguous images as more negative than males.

**Fig 3:**
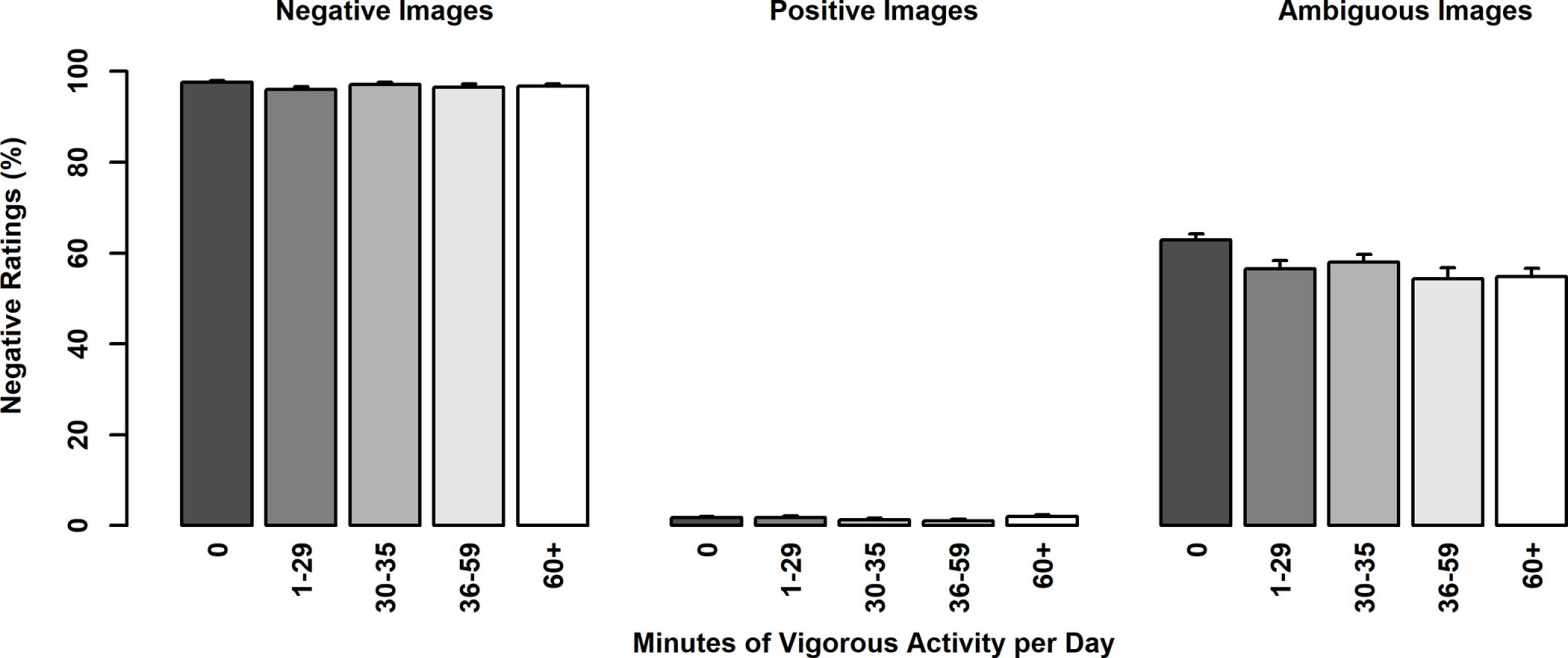
Percent negative ratings of emotional images as a function of vigorous activity in minutes per day. Ambiguous images were rated more positively by individuals that reported just a few minutes of vigorous activity per day as compared to those who reported no level of vigorous activity per day (p = .007).

Linear regression models with duration of vigorous PA grouped by minutes per day (5 duration levels of vigorous PA: 0 minutes, less than 30 minutes, 30–35 minutes, 36–59 minutes, and 60 or more minutes per day) significantly predicted ratings of ambiguous images (F(6, 473) = 4.50, p < .001). When examining effects between adjacent groups (i.e., comparing individuals who were inactive—0 minutes—to those who were active less than 30 minutes per day, then comparing less than 30 to 30–35 minutes, etc.), the only significant effects were observed between 0 and less than 30 minutes of vigorous activity per day and only for ratings of ambiguity (p = .007; Bonferroni corrected significance p < .013), such that those who engage in no vigorous PA were more negative (Fig 3). No other adjacent comparisons were significant (all p’s > .200). Again, an effect of sex revealed that females rated ambiguous images as more negative than males (p = .017).

### Association between valence ratings and exercise

There was a significant negative correlation between amount of exercise (hours per week) and ratings of ambiguously valenced images (r = -.157, p < .001; Bonferroni corrected significance p < .0167), such that more exercise was associated with more positive ratings of ambiguously valenced images. There was no such correlation with ratings of positive (r = -.064, p = .131) or negative (r = .016, p = .711) images.

## Discussion

The present findings provide evidence that engaging in more vigorous PA and exercise is associated with more positive interpretations of emotional ambiguity (i.e., a more positive valence bias). Specifically, this positive relationship with PA was linked to activity intensity, but also amount (frequency and duration), as individuals who conducted more vigorous PA, measured in minutes per week as well as minutes per day or days per week, demonstrated a more positive bias. With regards to exercise, more exercise hours per week were associated with a more positive valence bias. Indeed, this evidence for more positive interpretations of images was specific to ratings of ambiguous images, as there were no significant effects with ratings of clearly valenced (positive or negative) images. Further, when examining PA as a function of intensity and step-wise increases in amount, we found significant effects on ambiguous ratings only between 0 and 1 days per week of vigorous PA and between 0 and less than 30 minutes per day of vigorous PA. This suggests that the greatest impact of increasing vigorous PA by one unit (day/week or minutes/day) on ratings of ambiguity is in the difference between no vigorous activity and minimal activity.

These findings represent a novel contribution to the literature linking PA and psychological well-being, and are consistent with a broader literature relating PA and exercise to mental health (e.g., depression). For instance, others have shown that as little as one hour of exercise provides a protective effect from the future incidence of depression (a disorder that is characterized by a chronic negativity bias [57]); similarly, vigorous PA more efficiently reduces depressive symptoms when compared to moderate activity [58]. Notably, much of the work to date related to depression and psychological well-being has focused on beneficial outcomes of PA on depression and mood states [1, 59], self-reported measures (e.g., self-esteem, anxiety; [1]), and attention to clearly valenced (predominantly negative) emotional stimuli (e.g., threat-related words or angry faces; [60–62], but see [63]). Our approach contributes a more sensitive measure of psychological well-being: the valence bias, or valence ratings of images that could be equally interpreted as positive or negative; and we find here that these ambiguous images are more sensitive to the relationship with PA than are ratings of clearly valenced (positive and negative) images.

Interpretations of ambiguity can reveal important individual differences not only in emotion processing, but also in coping strategies (e.g., emotion regulation) that are crucial for mental health and social interactions. For example, our *initial negativity hypothesis* posits that the initial interpretation of ambiguity is negative ([25–26, 29] and that a positive valence bias relies at least somewhat on one’s ability to override that initial negativity via some emotion regulation mechanism [33]. One recent study has demonstrated that individuals with a positive valence bias show brain responses to surprised faces that overlap with responses during an explicit cognitive reappraisal task [33]. Further, both a positive valence bias [64] and enhanced reappraisal ability are associated with decreased depressive symptoms [65–66] and improved cognitive control [67].

Relatedly, PA and exercise are associated with enhanced emotion regulation success [43] and with lower symptoms of depression and anxiety [68–69], less persistent anxiety [70] and in some cases, may be an effective treatment for mild-to-moderate depression [71]. Taken together, interpretations of emotional ambiguity may represent a sensitive measure that could link PA and psychological well-being via a mechanism of emotion regulation, or reappraisal more specifically. In other words, emotion regulation (reappraisal ability) might serve as a mechanism by which PA promotes a more positive valence bias–for example, PA might enhance emotion regulation ability, which promotes more positive interpretations of ambiguity, but future work is needed to test this mechanism. Indeed, there are many mechanisms through which PA and exercise might alter emotional interpretations; while speculative, it is possible that PA and exercise affect neurotransmitter or neurotrophic factors, stress and hormonal pathways, or additional behaviors (e.g., sleep, eating) that may result in better overall brain health or improved cognitive control [24, 72]. Additionally, it may be that individuals with higher levels of cognitive control are better able to maintain plans and inhibit alternative behaviors to more regularly engage in PA, perhaps vigorous PA in particular, although this is likely a bidirectional relationship [73].

### The valence bias was related primarily to intensity, but also amount of PA

The relationship between PA and valence bias was tied to the activity intensity such that both component measures of amount (frequency and duration) of vigorous PA were significant predictors of bias; whereas moderate PA and walking did not have similar relationships with bias. These findings mirror reports demonstrating that the influence of PA on depressive symptoms also relies on activity intensity. In a cross-sectional analysis in over 12,000 adults, only vigorous PA was significantly associated with a reduction in depressive symptoms such that individuals who conducted no vigorous PA were 49% more likely to be depressed [58]. Although the study was conducted among Korean adults, the authors used the same guidelines to classify PA into vigorous, moderate, and walking as our study. To our knowledge, an association between moderate PA and depressive symptoms was only reported in older adults 65 years and older [74]. As participants in the present study represented a rather young sample with an average age of 32.6 years and only 2 individuals age 65 and older, future studies are needed to establish whether moderate intensity activity may have beneficial effects on valence bias in an older demographic.

Despite the correlative relationship between vigorous PA and positive valence bias, further exploration of these findings revealed that, as predicted, this effect appeared mostly independent of the amount (in days per week or minutes per day) of activity as long as participants conducted any activity at all. In other words, the greatest impact of increasing vigorous PA by one unit (day/week or minutes/day) on ratings of ambiguity is in the difference between no vigorous activity and minimal activity, such that individuals reporting even a minimal amount of vigorous activity showed a more positive bias than individuals reporting no amount of activity. While there may be cumulative effects (e.g., the difference between zero and one is smaller than zero and five days of activity), future work will be needed to more directly test these effects. Our results provide a more fine-grained analysis of activity amount than previous cross-sectional studies linking PA and depression in Korean adults, which used a threshold of 20 minutes of vigorous activity on at least three days per week to differentiate active from non-active individuals [58]. We suggest that the amount of activity needed to produce beneficial effects on emotional interpretations and psychological well-being could be less than the current Physical Activity Guidelines, which recommend at least 75 minutes of vigorous PA, at least three times a week [46]. This is consistent with reports of protective effects from depression with equally small amounts of exercise (1 hour per week) or PA (10 minutes per day [57]) and that happiness is greater in individuals who exercise at least once a week [44].

Finally, it is worth noting that, although the effects for vigorous PA appeared primarily as a function of intensity, the effects for exercise (a subset of PA) were associated with amount. Indeed, the various forms of exercise that we provided as examples (walking, jogging, running, stair climbing, aerobics, water aerobics, cycling, rowing, swimming, weight training, etc.) are predominantly in the vigorous category, based on their MET values [54]. However, it is certainly possible that some of these forms of exercise could include moderate intensity as well, especially in untrained and older individuals. Given that we did not collect data from these participants to specifically define the level of intensity for exercise, further exploration will be needed for examining the effect of intensity and amount of exercise on the valence interpretations of emotional ambiguity.

### Limitations

This study has several limitations. First, we did not collect information about height and weight from each participant. As such, we were unable to determine the extent to which our effects are modulated by weight status or body mass index. Although there is an established association between obesity and depression [75], the directionality remains under debate. Whereas some authors have argued that obesity may cause depression through negative body image [76], it has also been hypothesized that depression can result in weight gain due to altered eating and PA patterns [77]. Second, PA and exercise data were collected online using self-report measures. The IPAQ is a widely used instrument for the assessment of PA and has been validated numerous times against objective measures. In addition, a recent meta-analysis reported that overall validity was highest for vigorous activity [78]. Regardless, future studies could incorporate objective measures of activity, such as accelerometry (although this method does not allow differentiation between PA and exercise). Finally, as with much of the extant work examining the effects of PA on psychological well-being, this was a cross-sectional design comparing individuals that report varying degrees of PA and exercise. This is particularly important given that the relationship between PA, exercise and emotion regulation or well-being may very well be bidirectional in nature. For example, successful emotion regulation may lead to enhanced PA (e.g., greater endurance in the face of physical challenges [79]).

Future work will be aimed at using a longitudinal design to examine the effects of PA in individuals participating in exercise programs. This would allow us not only to have a more objective measure of PA over time, but to also compare ratings of ambiguity before and after participating in such a program, and even assess acute effects of PA on ratings (i.e., before and after the first session in the program). Indeed, previous work has shown that, although the valence bias is a stable, trait-like individual difference [28–29], a variety of experimental manipulations (e.g., stress, time perspectives, instructions to deliberate) are able to shift the valence bias, at least temporarily [25, 39, 80]. Notably, because our valence bias task does not constrain the valence of the participant’s response (positive or negative ratings of surprise are equally valid), it can be useful not only in determining the propensity of individuals to find positive meaning in ambiguity but can be used in future manipulations that train them to do so (e.g., promoting PA; see also 80). As a result, this approach offers a novel contribution to research on negativity bias using a new approach that controls the information perceived by the participants (i.e., viewing the same dual-valence images) and measures individual differences in emotion perception as a function of PA. Given the link between vigorous PA, exercise and a positive valence bias established in the current study, we would predict that participation in a controlled exercise program would produce a robust shift toward a more positive bias. This work should also include measures of depressive symptoms to directly probe relationships among depression, valence bias, and PA/exercise.

## Conclusions

The present findings represent the first evidence that vigorous PA and exercise are associated with more positive interpretations of emotional ambiguity (i.e., emotional information that could be interpreted as having either a positive or negative meaning). This link is particularly sensitive to the distinction between inactivity and any amount of vigorous PA (in terms of duration or frequency), as the strongest effects were evident in individuals completing any frequency or duration of vigorous PA compared to those completing none. Notably, although previous work has examined the relationship between PA and attention to clearly valenced (predominantly negative) emotional stimuli, this work represents an important extension of these findings by demonstrating that ambiguity, which allows for greater variability in interpretations, may be particularly well-suited for characterizing the link between PA and emotion. However, given the cross-sectional nature of these findings, future work should examine the longitudinal effects of PA on valence bias among individuals participating in a structured exercise program; in so doing, the valence bias measure could serve as a useful contribution to characterizing the beneficial effects of a physically active lifestyle.

## Supporting information

S1 Fig Percent negative ratings of emotional images. Each participant is represented along the x-axis, with ratings for angry (triangle), happy (square), and surprised (star) expressions. In other words, angry faces are rated as mostly negative, as evidenced by the triangles aligning the top of the graph, and happy as positive, as evidenced by the squares aligning the bottom of the graph. As is apparent here, there is much more variability in ratings of surprised faces than angry and happy. Even participants that were removed (e.g., one participant rated happy faces as negative on nearly 70% of trials) show some variability for clearly valence expressions, but not to the extent that there is variability for surprised faces.
(TIF)
