# Supplementary figures and images for: One step at a time: Physical activity is linked to positive interpretations of ambiguity
Source: PLoS One. 2019 Nov 14;14(11):e0225106. doi: 10.1371/journal.pone.0225106 (PMC6855442; doi:10.1371/journal.pone.0225106)

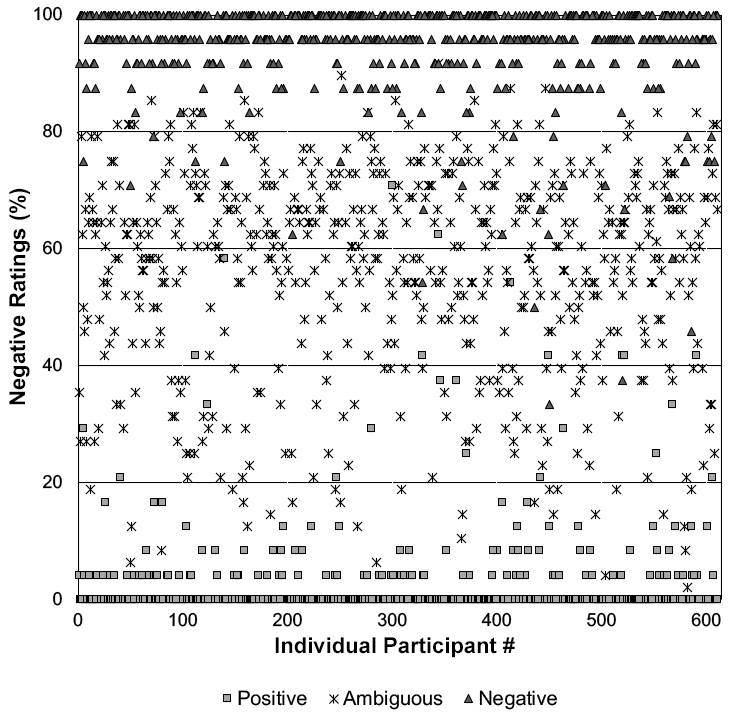

Supplement: S1 Fig — Each participant is represented along the x-axis, with ratings for angry (triangle), happy (square), and surprised (star) expressions. In other words, angry faces are rated as mostly negative, as evidenced by the triangles aligning the top of the graph, and happy as positive, as evidenced by the squares aligning the bottom of the graph. As is apparent here, there is much more variability in ratings of surprised faces than angry and happy. Even participants that were removed (e.g., one participant rated happy faces as negative on nearly 70% of trials) show some variability for clearly valence expressions, but not to the extent that there is variability for surprised faces. (TIF) [file pone.0225106.s001.tif]
